# Parents’ perspectives on preparing for parenthood: a qualitative study on Greenland’s universal parenting programme MANU 0–1 year

**DOI:** 10.1186/s12884-022-05170-4

**Published:** 2022-11-20

**Authors:** Christine Ingemann, Else Jensen, Ingelise Olesen, Tine Tjørnhøj-Thomsen, Siv Kvernmo, Christina Viskum Lytken Larsen

**Affiliations:** 1 Centre for Public Health in Greenland, National Institute of Public Health, University of Southern Denmark, Studiestræde 6, 1455 Copenhagen, Denmark; 2 Greenland Center for Health Research, Institute of Health and Nature, Ilisimatusarfik – University of Greenland, Nuuk, Greenland; 3 Department of Health and Social Context, National Institute of Public Health, University of Southern Denmark, Studiestræde 6, 1455 Copenhagen, Denmark; 4 IKM, Faculty of Health Sciences, UiT – The Arctic University of Norway, Tromsø, Norway

**Keywords:** User perspective, Parenting program, Parent education, Preparation, Implementation, Arctic, Circumpolar, Qualitative methods, Indigenous perspective, Thematic analysis

## Abstract

**Background:**

The transition to parenthood has received increasing attention in research, partly due to evidence pointing out the crucial developmental period of a child’s first thousand days. Parenting programmes aim to prepare and support families in their transition and distress. For a programme to be implemented successfully it is important to consider parents’ needs and resources. Bringing parents’ perspectives and experiences to the forefront of the implementation of the Greenlandic parenting programme MANU 0–1 Year (MANU) is important for determining if the programme can meet its aim of contributing to thriving families. This study aims to investigate how parents’ notions and experiences of parenthood are reflected and challenged in MANU.

**Method:**

Data were collected in three of Greenland’s five municipalities. Qualitative interviews were held with 38 mothers and 12 fathers either individually or as couples: a total of 40 interviews. Additionally, a Sharing Circle with three fathers was held. Interviews were in Greenlandic or Danish. A thematic, inductive analysis was applied.

**Results:**

In their transition to parenthood, participants experienced a reprioritisation of their life and changes in their network. It is important to parents that their child experiences security and care, and participants describe this in contrast to their own childhood. Community is the most important value in child-rearing. Conversations and advice from family members and friends are mentioned as a means to prepare for birth and parenthood. Additionally, conversations with midwives and MANU sessions were also used for preparation. Parents appreciated learning from and listening to other parents in MANU sessions. However, accessing MANU depends on the individual parent’s interest and ability to attend sessions.

**Conclusions:**

Parents’ notions and experiences of parenthood are addressed in the programme, but the use of MANU depends on the parents’ attendance and how it is organised and locally offered. The study suggests that MANU has the possibility to create a space for parents to reflect and prepare. However, for MANU to be universal as intended and to reach both mother and father the facilitation of sessions could be revisited.

**Supplementary Information:**

The online version contains supplementary material available at 10.1186/s12884-022-05170-4.

## Background

In recent years, there has been increasing public and scholarly attention to the transition to parenthood. This is partly based on evidence showing that from the prenatal period until the age of two is a crucial development period for the child. This period is called ‘the first thousand days’ and is considered critical for shaping the foundation for the child’s health and development [1]. Parents’ choices in parenting and upbringing influence the health of their children throughout the child’s development both physically and mentally [2]. Additionally, the transition to parenthood is known to be a major developmental period for families while learning to care for a newborn [3–5]. This period increases relationship stress and disharmony due to the new responsibilities and negative outcomes of unfulfilled expectations after childbirth [5, 6]. Besides parents needing emotional and psychological support during this transition [6], evidence also strongly recommends that families receive guidance for providing nurturing care and protection so that children achieve their developmental potential [7].

Parent education programmes targeting either specific at-risk sub-populations or those designed as universal programmes accessible to everyone are developed internationally [3, 8, 9]. An underlying hypothesis of many parenting programmes is that knowledge deficit among parents is a major cause of parental distress [10]. Furthermore, parents’ knowledge and needs are barely explored prior to programme development. Universal programmes may be of value when parents can access the programme at a time and in a format that suits them [8, 10].

The universal Greenlandic parenting programme MANU 0–1 Year (MANU), which stands for *Meeraq Angajoqqaat Nuannaarneq*, meaning ‘child’s and parent’s happiness’, was developed to provide expectant and new parents with relevant information and reflections on how they want to be as parents through pedagogical exercises [11, 12]. MANU is based on developmental theories, international evidence on the first thousand days and the high number of vulnerable families in Greenland [11]. Ultimately, MANU is expected to secure a healthy foundation for children’s development and to contribute to the prevention of adverse childhood experiences [12, 13].

Previous studies in Greenland show the importance of appropriately integrating cultural beliefs, values and local practices into Greenland’s public health and healthcare system [14–16]. Cultural values and Indigenous knowledge are fundamental in parenting programmes in populations comparable to Greenland. These include the breastfeeding initiative built on traditional infant feeding practices in the Northwest Territories of Canada [17, 18], and Indigenous-led parenting programmes like the First 1000 Days Australia [19] and the Inunnguiniq parenting programme in Nunavut, Canada [20, 21].

MANU is offered as a universal programme, but it is uncertain how the programme meets the needs of Greenlandic parents in general. Since only few perspectives of parents were explored during the development process of MANU. Considering parents’ needs, expectations, attitudes and resources are important determinants in programme development and implementation [22–24]. Bringing parents’ perspectives and experiences to the forefront of MANU’s implementation process is valuable for understanding what hinders or supports programme implementation as well as if the programme has the potential to meet the ultimate outcome of healthy and thriving families [11, 22]. Therefore, this study aims to investigate how parents’ notions and experiences of parenthood are reflected and challenged in MANU: first, by investigating parents’ experiences of their transition to parenthood and identifying their values in child-rearing; then, by comparing these experiences with MANU’s content and parents’ perspectives on attending MANU to identify opportunities to advance the programme to meet parents’ needs.

## Methods

This study applied qualitative methods to investigate parents’ perspectives on parenthood in relation to the parenting programme MANU. The Consolidated Criteria for Reporting Qualitative Research (COREQ) were applied for reporting on study design, analysis and findings, which are domains 2 and 3 in the checklist [25]. The present study is part of a PhD project which applies a community-based participatory research (CBPR) approach. Based on N Wallerstein, B Duran, JG Oetzel and M Minkler [26] five stages in CBPR, a reference group consisting of stakeholders involved or related to MANU was established at the very beginning of the PhD project [11]. Furthermore, in the present study the reference group set the study’s aim, decided on study sites, advised on interview questions, discussed use of a translator versus a Greenlandic interviewer, contributed to the analysis and engaged in recommendation development. Scholars have increasingly discussed the importance of applying approaches like CBPR in circumpolar health research [27, 28]. In this way, the study and its results are placed back in the hands of the participants themselves, who own and need the knowledge.

### Research setting and MANU

*Kalaallit Nunaat* is the Greenlandic name for Greenland and can be translated as ‘Land of the people’ or the ‘Land of the Kalaallit’ [29]. Most of the Greenlandic population, close to 90%, are ethnic Greenlanders (Kalaaleq/Inuit) – a recognised Indigenous population. Most Greenlanders speak the national language *Kalaallisut* (Greenlandic), while both Danish and Kalaallisut are taught in schools. Greenland is the largest island and least densely populated country in the world, with a total population of 56,421 and a fertility rate of 2.1 in 2021 [30]. Towns and settlements are isolated from each other, meaning they are only reachable by air or sea. About 60% of the inhabitants live in one of the five regional towns, and the remaining population lives in the other 11 towns and about 54 settlements. The regional hospitals are located in the five regional towns. Women can give birth in the national hospital in Nuuk (capital city), the regional hospitals in Sisimiut and Ilulissat or Tasiilaq’s health centre. Countrywide, there are marked socioeconomic and infrastructural differences between towns and settlements [31]. Greenland is a former Danish colony, which gained Home Rule in 1979 and Self Rule in 2009 but is still part of the Kingdom of Denmark. Greenland has roughly adopted the Danish welfare-state model and healthcare system. Since 1992, the healthcare system has been fully administered by the Greenlandic government.

### Study sites

Three of the five municipalities in Greenland were selected for this study. Data were primarily collected in the regional towns of these municipalities, while a few smaller towns were included through phone interviews or if a participant was visiting the regional hospital due to childbirth. In 2020, the researchers spent two weeks on the first site and three weeks on the second site. On the third site, data collection was spread out over a longer time period due to the first (CI) and second (EJ) author living there.

### The parenting programme MANU

Since the development of the parenting program MANU 0–1 Year in 2016, a range of other MANU programme items have been developed [11]. This study has focused on MANU’s first item ‘MANU 0–1 Year’, which aims to prepare expectant parents for parenthood. MANU 0–1 Year, henceforth MANU, provide parents with a book containing information and conversational exercises, and six antenatal and three postnatal 2.5-hour sessions provided by midwives, public health nurses (Danish: *sundhedsplejerske*), or health assistants as facilitators [11, 12]. The book and sessions coincide in terms of content. Parents are provided with information on the pregnancy progress, both physical and mental; healthy nutrition, including the harm of consuming alcohol and hashish; the development of the child’s brain; signs of birth and birthing phases; a newborn’s needs; and the importance of care for the child’s development. MANU addresses topics on bonding and sensitivity; having to travel to give birth; sex during and after pregnancy; practical preparations for the child’s arrival; sibling jealousy; a typical day with a newborn; family network; reflections on one’s own childhood; setting boundaries; and opportunities and challenges associated with receiving support and advice from elders. Connected to this information and the addressed topics, there are pedagogical exercises inviting parents to reflect on their own thoughts and share their thoughts with each other.

The first two years of MANU’s implementation focused on disseminating material and training professionals on a three-day introductory course [11]. In the programme’s early stage of implementation, local challenges with carrying out MANU were already anticipated or experienced by health professionals, and adaptations were made to the frequency of group sessions offered [11]. Furthermore, during the COVID-19 pandemic, MANU group sessions were not offered due to restrictions on group gatherings. No alternative versions of MANU were offered during lockdowns.

### Data collection

#### Interview guide

The interview guide was developed by CI based on topics and questions relating to: i) child-rearing and parenthood in MANU [12]; ii) topics discussed with the reference group and with the author team based on their fields of experience and implementation science [22, 23]; and iii) an explorative pilot interview in Danish with a parent. The translation of the interview guide was an iterative process between EJ, IO and CI, in which understanding and wording of the questions were discussed, as well as the structure of the interview guide. After a second pilot and the first interviews were conducted, the last few alterations to the guide were made.

The interview guide was divided into two topics: i) family and parenthood and ii) MANU. An English version of the interview guide is provided in Additional file 1: Appendix 1. As an introduction, participants were asked their age, where they grew up and whether it was their and their partner’s first child. Questions in the first part of the interview were on pregnancy decision; life changes in connection to parenthood; values in child-rearing; important family relations in child-rearing (using a blank circle diagram for illustration, see Additional file 2: Appendix 2); responsibility in child-rearing; reflections on parents’ own childhood experiences and connections to the older generation (e.g. grandparents); and who participants get advice from and on what. The second part on experiences with MANU was developed based on parents’ preferences, expectations, attitudes, knowledge, needs and resources, which can influence implementation [22]. Questions concerned if and how participants were invited to take part in MANU; how often they attended, and if not why; if their partner attended; if any topics were of specific interest to them; how they liked the set-up of the MANU sessions; if it met their expectations; and if they had any suggestions.

#### Recruitment of participants

The study set out aiming to interview six couples on each of the three sites: four couples who had attended the MANU parenting programme and two who had not. Furthermore, criteria for participation were that parents should either be pregnant or their youngest child should not be older than 1 year. In agreement with the local midwives and public health nurses, participants were recruited in the waiting room for midwife or public health nurse consultations, at the hospital ward, patient hotel, from participant lists of MANU sessions and at observed MANU sessions. Additionally, participants were recruited outside the health centre or on the street and through posts in relevant local Facebook groups and small posters in towns. Potential participants were primarily approached and invited by EJ in Greenlandic.

#### Semi-structured interviews

A semi-structured interview design was chosen, since the study is interested in parents’ perspectives on specific themes, namely parenthood, child-rearing and MANU [32]. Participants could choose to be interviewed in Greenlandic or Danish. The semi-structured interviews were conducted primarily in Greenlandic by EJ, while CI was present during all interviews to observe the conversation and be available for potential questions. Participants were interviewed either in CI’s home or office, or the participant’s home.

#### Sharing circle with fathers

Sharing Circles, an Indigenous form of communication [33], were used as a data collection method for the sharing of experiences [34]. In an attempt to include more male participants in the study, the authors tried to organise Sharing Circles on two sites, but this only succeeded at one site. At the first Sharing Circle only one out of five invited fathers came; he was individually interviewed. At the second Sharing Circle, three out of six invited fathers participated.

For the second Sharing Circle, a community room was provided by the municipality. The room was a broad airy space, where chairs were placed in a circle in the middle of the room. Refreshments were provided, and participants gathered and greeted each other. Then participants took a seat. CI stayed outside the circle for observation. EJ facilitated the Sharing Circle in Greenlandic. First, the study and the day’s topic were introduced, then EJ introduced the rules of the Sharing Circle. These included ethical considerations of confidentiality, that the person who is talking should not be interrupted and that everyone would be given the opportunity to share. First, participants were asked about their reaction when they found out they would become a father; then values that were important to them in child-rearing; who, besides them as parents, plays an important role in raising their child; who they ask for advice regarding their child or parenthood; and how they had prepared for parenthood and if they had missed anything.

#### Field notes

In order to keep track of the persons recruited, field notes were taken during data collection. Furthermore, during the Greenlandic interviews CI took participatory notes on the flow and atmosphere of the interview [35]. Consolidated field notes were taken after each interview; CI and EJ briefly reflected on their experience and their daily experiences of the data collection process [35]. These notes were used to recall and understand the context the interviews were held in.

### Study participants

Qualitative interviews were held with 38 mothers and 12 fathers, either individually or as couples. The interviews had a duration of 25 to 50 minutes, and the Sharing Circle with three fathers took about 1 h. Table 1 provides an overview of the interviews with an indication of the language, Greenlandic (GL) or Danish (DK), couple or individual and if the participants are from a large or small community.

**Table 1:** Overview of interviews

Site	Size of town	Couple interviews	Individual female interviews	Individual male interviews	Sharing Circle with fathers
A	Regional town	1 in GL	4 in GL 2 in DK	2 in GL	/
	Small town	/	3 in GL	/	/
B	Regional town	3 in GL 1 in DK	8 in GL	/	1 in GL with 3
	Small town	1 in GL	1 in GL	/	/
C	Regional town	4 in DK	3 in GL 5 in DK	/	/
	Small town	/	2 in GL	/	/

GL indicating interview held in Greenlandic and DK in Danish

Fewer couple interviews were held than initially planned. A total of 30 people declined, cancelled or did not attend the scheduled interview. Men were often not interested in participating or could not find the time due to work. When recruiting, it was difficult to identify if parents had participated in MANU fully, to some degree or not at all. Table 2 provides an overview of participants’ age, their newborn’s age or if they were pregnant at the time of the interview, if they have older children, if they are partners with the mother or father of their newborn child and if they have attended any MANU sessions.

**Table 2:** Overview of participant characteristics

Site	A	B	C
Total number of participants	13	22	18
Participants’ age	21–25: 5	21–25: 7	21–25: 3
	26–30: 1	26–30: 5	26–30: 8
	31–35: 5	31–35: 6	31–35: 3
	> 35: 1	> 35: 1	> 35: 3
	Unknown: 1	Unknown: 3	Unknown: 1
Age of newborn or pregnant at point of interview	Pregnant: 2	Pregnant: 5	Pregnant: 7
	1–6 months: 5	1–6 months: 9	1–6 months: 9
	7–12 months: 5	7–12 months: 6	7–12 months: 2
	13–15 months: 1	13–15 months: 1 Unknown: 1	13–15 months: 0
Older children?	5 Yes	13 Yes	7 Yes
	8 No	9 No	11 No
Together with the newborn’s father/mother?	12 Yes	22 Yes	14 Yes
	1 No	0 No	3 No
Attended MANU sessions	9 Yes	13 Yes	17 Yes
	4 No	9 No	1 No

Parents who had attended MANU sessions could rarely remember the precise number or which one of the nine different MANU sessions they had attended, therefore it is not possible to provide a reliable number of sessions attended by study participants.

### Data analysis

Interviews were audio-recorded after obtaining participants’ consent. Then, recordings of the Danish interviews were transcribed primarily by CI and also by a Danish student assistant. The Greenlandic interviews were primarily transcribed and translated by EJ and also by two Greenlandic student assistants.

A thematic, inductive analysis of the transcriptions and fieldnotes was performed [36]. All data were imported into the qualitative data analysis software NVivo12. First, randomly selected transcripts were thoroughly read by CI, EJ and IO. Then, general themes and impressions from these transcripts were discussed. As a next step, CI coded all transcripts in NVivo, based on the themes discussed in the first step. Thirdly, themes were summarised, and some themes were re-coded. Lastly, transcripts, themes and summaries were revisited for validation of the presented results.

The quotes presented in this paper were translated from Kalaallisut to Danish to English, or from Danish to English.

### Ethical considerations

The study has been performed in accordance with the Declaration of Helsinki, and the Greenlandic Scientific Ethical Committee (Danish: *Videnskabsetisk Udvalg*) granted ethical approval of the project.

Each participant was provided with an informed consent form in either Danish or Greenlandic, and the content was explained. Participants signed the form to document their informed agreement and received a copy with the researcher’s contact details. For the de-personification of the data, codes for each participant and material were developed. An Excel spreadsheet was used as a tracking method to keep an overview of planned, held and cancelled interviews. The date, participant’s contact details, length of interview, method of recruitment, interviewer, transcriber and quality check of transcription were noted. All data are stored on an encrypted drive, in accordance with data management guidelines. All stakeholders and participants were invited to receive an e-mail newsletter on updates and results of the study.

The author team reflected on potential ethical concerns prior to interviewing couples and addressing parenthood, child-rearing and own childhood experiences. When interviewing couples, the relation between the two participants might influence or limit their responses, but it can also, on the contrary, enhance them. However, this was difficult to determine during the interviews, as the interviewer was with participants for such a short period of time. Furthermore, the addressed interview topics are sensitive in that they are connected to a person’s identity, opinion and own experiences. Additionally, parenthood and child-rearing are topics widely discussed in society about which the public had many opinions on what is right and wrong [32]. A few participants became emotional, for example, when asked about the changes they have experienced in their transition to parenthood. Nonetheless, at the end of the interview they were thankful for the opportunity to reflect on their experiences and to express their thoughts.

## Results

**Fig. 1:**
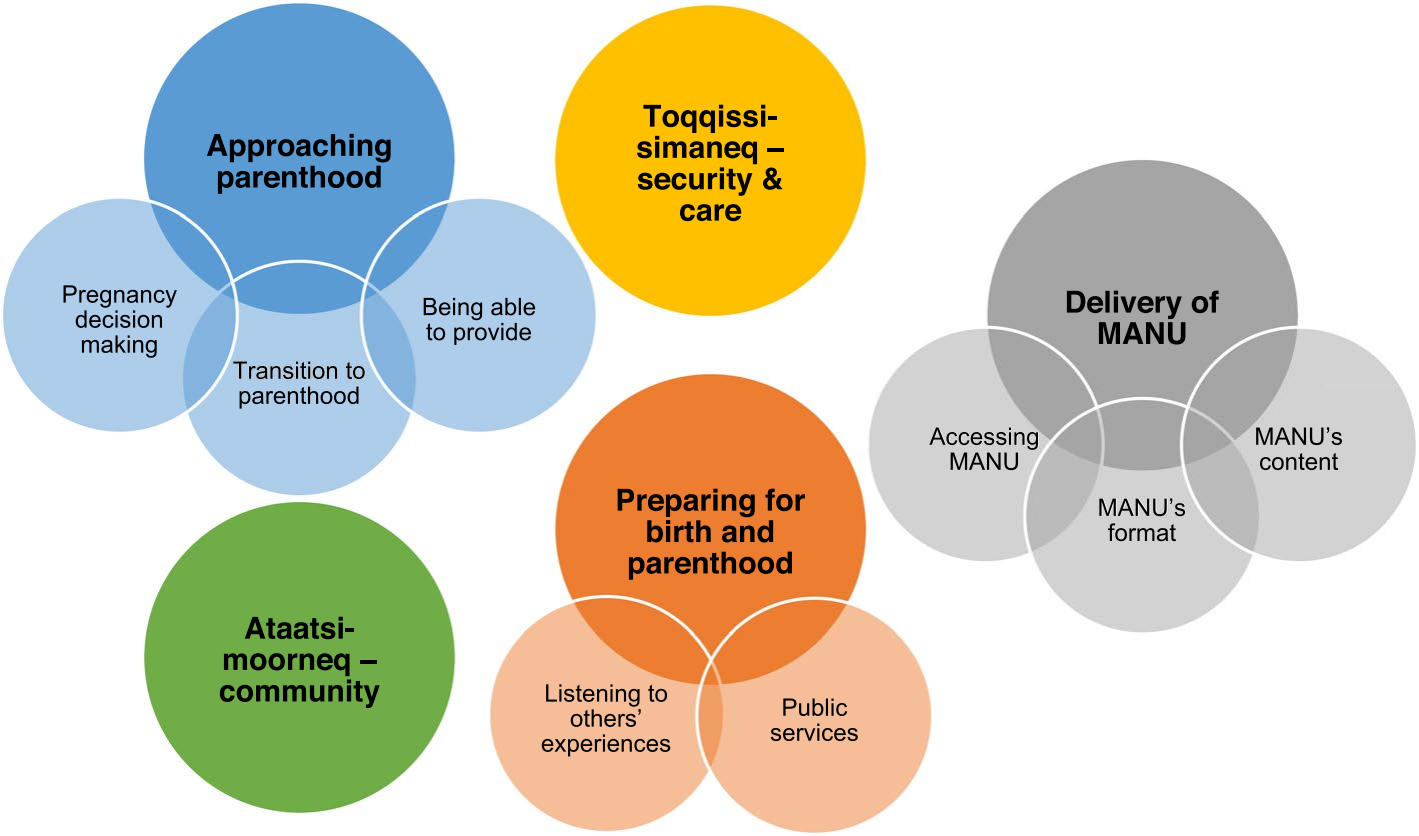
Overview of themes and sub-themes

The study’s results are organised into the following themes and sub-themes: (i) approaching parenthood, (ii) toqqissisimaneq – security and care, (iii) ataatsimoorneq – community, (iv) preparing for birth and parenthood, and (v) delivery of MANU. Some of the themes contain further sub-themes, which are presented in Fig. 1. The first four themes show how parents experience their transition to parenthood, prepare for it and how they understand child-rearing, while the fifth theme focuses specifically on parents’ experiences with attending the MANU programme.

### Approaching parenthood

In parents’ approaches to parenthood, their first step was deciding to be pregnant. Then, in their transition to parenthood they experienced changes in their lives and wanted to provide for their unborn child.

#### Pregnancy decision making

Participants had both similar as well as different starting points to parenthood. Many of the participants had at some point in their relationship with the co-parent discussed their desire to have children, as one participant expressed: “We didn’t specifically decide it, we just welcomed them (children), when they arrived (were conceived)” (Mother 1). The decision to become parents often related to taking a step forward in life and the wish to create one’s own family. Participants also described how having a child is an act of love and connected to the wish to create a happy family, and one mother expressed how having a child is about “love and that it should go well as a family” (Mother 2). However, for some participants the pregnancy was unexpected in terms of not knowing whether to keep or abort the pregnancy, and a few found out too late to even consider an abortion. For them, the support they received from their partner and parents, as well as the emotional bond they developed with the child contributed to their decision to keep the unborn child.

#### Transition to parenthood

Regardless of whether the pregnancy was expected or not, the transition to parenthood was a great upheaval for the participants. They described how becoming a parent is lifechanging. It reshaped their perspective on their surroundings, changed their priorities, affected their friendships, and shifted their focus from themselves to their child. One father expressed how he can no longer imagine being without his daughter:

“Now my happiness is no longer out in nature, in solitude. It is in my daughter. I won’t be able to go out into nature alone as before. To go out for a walk alone. It may never be like that again. If I'm going out into the nature today, it has to be together with my daughter.” (Father 2)

Many defined the transition to parenthood as leaving their youth and freedom behind, while receiving a lifelong responsibility; one participant described her experience: “You get a huge responsibility after the birth, which you have the rest of your life. That was perhaps the most overwhelming thing for me” (Mother 4). Many parents expressed they have had made lifestyle changes by limiting or refraining from alcohol consumption, or, for some, getting out of their alcohol or hashish addiction, when they found out they were to become parents. Stepping away from addiction and youth life gave both fathers and mothers confidence and the desire to become independent, find a job and get an education. As one emphasised: “Before I had children, I never thought of taking an education” (Mother 5). Leaving youth life behind meant giving up partying and visiting cafés, which decreased participants’ friendships, though the real and close friends remained. Similar to others, one mother explained how becoming a mother was everything she desired, but after having a child she was surprised by how tough it is mentally to put your child’s needs before your own.

#### Being able to provide

With the transition also came the desire to be able to economically provide for the child. Many participants expressed that they did not want their child to lack anything. For participants, the most important preparation was to buy the necessary practical things for the child’s arrival (e.g. clothes, pram). Furthermore, many discussed the importance of having or finding a job, finishing their education and moving out of their parents’ or their partner’s parents’ house. However, finding a place to live is a matter of joining long waiting lists for an affordable apartment, due to lack of housing in many communities in Greenland. One father described how him and his partner are waiting for a flat that is suitable for their newborn:

“It takes a long time. There are only flats available near [area]. It is not suitable to live there when you have a child. There is drinking and quarrels. It is the only apartment block they offer, but we do not want to live there.” (Father 3)

Despite appreciating their parents’ support, it is not perceived as ideal; as one mother puts it: “It is strange to imagine that we will become a family while still living with his parents.” (Mother 6).

### Toqqissisimaneq – security and care

*Toqqissisimaneq* is Greenlandic (Danish: *tryghed*) and can be translated to security and care. This second theme mainly stems from the responses to the interview questions: ‘How do you want to see your child grow up? Which values do you see important in child-rearing?’ and ‘What do you want to do differently compared to your own childhood and why?’ When parents were asked to define toqqissisimaneq, several aspects were connected with the term. Most participants defined it as providing a secure environment that is free of alcohol, hashish and violence, but it also includes a calm home, stability, prioritising the child’s needs and being without worries. Participants want to achieve this by giving love, creating close (body) contact and ensuring the child feels cared for and is able to discuss difficult matters.

Participants shared how they went through adversities connected to alcohol, hashish and quarrels in their childhood home. In the following quote, one father expressed how hard it is to experience this in one’s childhood:

“It's because it's the worst thing you experience in your childhood. When parents get drunk, they change completely. It's the worst experience you can have. […] It is harsh, and you feel apprehensive.” (Father 2)

Based on these experiences and the desire to prevent adversities, participants have mostly banned alcohol from their home and saw it as important for their child to not experience alcohol in their upbringing. As one mother clearly pointed out: “I do not want to pass this on to a child.” (Mother 16).

Knowing how to care for one’s children and collaborating as parents is important, which participants reported in the light of having experienced the opposite in their childhood. Parents wanted to create a stable home with routines and boundaries, where their children learn the importance of going to school, being independent, gaining self-confidence and learning to be open and curious about life. In this respect, one mother described the importance of boundaries:

"I want to teach my child to set boundaries and that he knows where his own boundaries are. And he should learn to be responsible." (Mother 9)

Good communication and respect were also important values, and parents wanted to show patience and explain decisions made. Furthermore, their child should be treated as an equal, feel heard and have a certain degree of influence on decision making. Respecting the child for who they are and supporting their interests was as important as teaching them to respect others, especially elders.

### Ataatsimoorneq – community

The third theme ataatsimoorneq arose from multiple responses to interview questions, where participants continuously mentioned and discussed the role of family and spending time together. Ataatsimoorneq is Greenlandic (Danish: *fællesskab*) and means community – sense of togetherness. Experiencing community or togetherness with extended family by learning about family ties, attending family gatherings and eating together on a regular or even daily basis when living in the same place stood out as an important value in child-rearing. Community is something participants had experienced themselves in their childhood, which they wanted their child to experience as well. As one mother expressed:

“Being together, for example eating together, community, and visiting each other. It is about having a good time together and laughing together.” (Mother 15)

Enjoying nature with family by going sailing, picking berries or going hunting is also valued by participants, but sailing and hunting can be an unaffordable expense. Even if extended family does not live in the same community, it is important for them to follow the child’s development through text messages or video calls.

Participants reported that grandparents had the closest relation to their child after themselves, even when the grandparents did not live in the same community. While parents agreed that it is primarily their responsibility to raise their child, some found that extended family (e.g. grandparents) can take part in it as well. Participants themselves remember the unconditional love and joy they received when spending time with their own grandparents in their childhood. This is something parents want their children to experience, as one mother described:” You feel great joy when you are with your grandparents. I would very much like to pass this on” (Mother 8).

### Preparing for birth and parenthood

The fourth theme deals with parents’ perspectives on preparing for birth and parenthood. Participants reported that they prepared by (i) listening to others’ experiences and (ii) using public services. These two sub-themes included parents’ experiences with MANU and will be elaborated below.

#### Listening to others’ experiences

The most common way to prepare for birth and parenthood for participants was by talking to their own mother, sister, aunt or friend who had experienced childbirth and being a parent. One woman described how she prepared to give birth:

“My mother has been very important. For example, I can ask her about birth, also my grandmother. Those who have experienced giving birth. That is how I have prepared myself to give birth.” (Mother 7)

Family members and friends are also an important source for advice and guidance after birth, when doubts arise as to the well-being and raising of the child.

When participants who had attended MANU sessions described their experiences with MANU, many valued the possibility to learn from the other expectant parents in their group. This way they could also learn from others’ experiences and perspectives on the topics addressed in MANU. One mother expressed her positive experience:

“It was good to meet other expectant parents, also those who already have parenting experience. You feel very welcome, and I did not feel any limitations on what I could share with the group.” (Mother 3)

Talking about thoughts and feelings as a couple regarding pregnancy, parenthood, their unborn child or relationship was for some participants a natural part of their relationship, while for others these conversations were rare or new. Men in particular described themselves and were described by women as quiet and having difficulty expressing themselves. From the interviews with men, it appears that they seldom sought advice and guidance in their network. However, they expressed a desire to talk to other fathers in similar situations about their experiences. In the following two quotes, two fathers expressed this need:

“I’ve missed talking to other fathers a lot for a while, being able to contact other fathers who have children at my own daughter's age. Just to be able to share with each other. I have missed that a lot. But we men are not always open. This is a challenge. It is not possible to contact anyone. Some days I do not feel like I have anyone I can contact, even though I have a large family and my partner.” (Father 1)

“That's also why I've said yes to participating in your study, so that I can get rid of my thoughts a bit. […] It would have helped a lot, if I had had the opportunity to talk about my thoughts as a father earlier on. In all areas it would have been very different for me. […] It is only recently, when I heard about you wanting to interview, that I came to think of how I haven’t had conversations at all. From the very first day she was born, it is now for the first time that I share my thoughts.” (Father 2)

In relation to this, they suggested that having a fathers’ group after birth, just like mothers meet in groups after birth, could be useful.

#### Public services

Preparational and supporting services and programmes from the healthcare system and municipality were described as important and useful by the study’s participants. Conversations with the midwife during consultation hours were central, and participants appreciated being able to contact health personnel at any time when needed during their pregnancy. One mother expressed how her relation to and confidence in her midwife was important during her pregnancy:

“The midwives have helped me a lot by talking with me about my anxiety. […] I felt understood […] But at one point during my pregnancy there was a high turnover in personnel, so I grew tired of having to explain everything from the beginning to the new staff.” (Mother 8)

After the birth, the home visits from public health nurses were appreciated, and participants welcomed the possibility to ask all kinds of questions they had regarding their newborn. Participants used the municipalities’ family centres to receive help, for example, regarding relationship conflicts, addiction or anxiety.

Parents, who had attended MANU sessions, were satisfied with the programme. As one mother described it: “MANU is a good thing, and I think all pregnant women should participate in it. With MANU one does not feel alone or confused” (Mother 9). One father reported how MANU helped them prepare for parenthood as a couple:

“It has helped us a lot in our preparation. Our conversations in MANU are different than at home. And the fact that the two of us can prepare together, that is something I have been very happy about.” (Father 1)

### Delivery of MANU

MANU was generally appreciated by participants, but many experienced challenges with accessing MANU. This fifth theme is divided into the following three sub-themes: (i) accessing MANU, (ii) MANU’s format, and (iii) MANU’s content.

#### Accessing MANU

Almost all participants were offered the opportunity to attend MANU sessions. Most parents received MANU group sessions, while parents with an addiction problem received MANU in individual consultations. Few participants recalled having attended all nine sessions. Others only attended a few sessions due to work, illness or other personal hindrances, as one mother described:

“I would have participated if it wasn’t during working hours. And, while I was pregnant, I wanted to gather more hours at work in order to be eligible for more maternity leave support. I didn’t want to lose any hours at work.” (Mother 8)

Men in particular did not attend MANU due to it being during work hours, in addition to lacking interest in MANU, as one father explained: “I was a bit indifferent to attending MANU. I just thought that we can handle it ourselves. But now after having attended MANU, I think it helps me” (Father 4).

MANU sessions were not always offered as the programme suggested, due to restrictions in relation to the COVID-19 pandemic, illness among personnel or too few people signing up. Furthermore, participants from smaller towns, where MANU was not locally delivered, did not know of MANU or were only able to attend the programme when waiting for childbirth in the regional or national hospital.

#### MANU’s format

As a result of many not attending MANU sessions due to hindrances, some participants described how this influenced group dynamics because they were too few or the parents attending changed with every session. In MANU group sessions, the facilitator (e.g. a midwife) used a slideshow to provide information, conversational exercises from the MANU book and invited parents to ask questions. Participants were generally content with this format, however, the facilitator’s motivation and skills to hold a session were noticed and deemed relevant to whether they got something out of it. This also influenced whether parents found a session of 2.5 hours too long or just right. Furthermore, one father expressed that he would have appreciated the sessions to be more physically active, since sitting down for 2.5 hours can be tiring, despite the information and topics discussed being interesting. Participants barely read the MANU book, except when using it during the sessions.

#### MANU’s content

During pregnancy, female participants expressed being very occupied with preparing themselves for birth. This was also frequently mentioned topic and session attended when interviewing parents about their experiences with MANU. Other topics parents remember from the sessions they attended varied, but they valued receiving information about how a newborn sleeps and eats, how to learn to distinguish its cries and what parents can do. Furthermore, they described having reflected on their own childhood and how they would like to be as a parent, and they also became aware of who was in their network. Having a space to have these conversations or just having these topics addressed was appreciated. The following two quotes describe the positive experiences with the exercises in the MANU sessions: “All the exercises were so good. They help us get to know each other better as a couple” (Mother 10) and “I feel the exercises trigger thoughts in my head” (Mother 11). Some participants would have liked to have received more guidance on how to nurture their relationship with their partner after birth. Many expected to receive more hands-on preparation in the MANU sessions, for example, on how to bathe or hold a baby. A few mothers mention that their male partner found MANU too theoretical and too much about feelings.

Many participants also sought information from books, the internet, Facebook groups (local groups for e.g. expectant mothers), smartphone applications (e.g. foetus development) and podcasts, such as the Greenlandic podcast on birth experiences ‘Ernineq’ (birth).

## Discussion

This study aimed to investigate how parents’ notions and experiences of parenthood are reflected and challenged in MANU. Participants described in qualitative interviews and a Sharing Circle how they experienced a reprioritisation of their life in the transition to parenthood, for example, by having to set aside their own needs and rearranging their network. It was important to parents that their child experiences security and care, and some participants described this in relation to their own childhood. Through good co-parenting and setting boundaries, parents wished to raise their children to be respectful, confident and independent. After parents, grandparents were the most important familial relation to the child. In connection to this, community and togetherness stood out as the most important value in child-rearing. Conversations, advice and guidance from family members and friends were most often mentioned as a means to prepare for birth and parenthood. Additionally, conversations with midwives and MANU sessions were also used in preparation. Besides the relevant information and topics addressed in MANU, parents appreciated when they could learn from and listen to other parents. However, accessing MANU depended on the individual parent’s interest and abilities (e.g. in relation to work or personal hindrances) to attend sessions and read the MANU book.

### A balance between awaiting and preparing

A period of unpredictability follows pregnancy and the transitioning to parenthood. Many participants described how the decision to become pregnant was unplanned, and only when they found out did they make the decision to keep the child, based on their current situation and their desire to have a child. MM Schlütter [37] discusses motherhood and unpredictability in an anthropological study conducted in Nuuk, Greenland. She identifies mothers’ approaches to the unpredicted events that come with pregnancy and parenthood as a Greenlandic narrative, in which the focus is on meeting life’s challenges with calmness and a sense of awaiting [37]. Accepting one’s situation as it comes and awaiting the unpredictable changes are common in an Arctic context, where Inuit people live close to nature. Greenlanders are often described as not planning far ahead or predicting different outcomes of a situation [37, 38], which stands in contrast to the external influence of urbanisation by which Greenlanders find themselves both resting in unpredictability while also adopting Western ways of having expectations and predicting outcomes. As an Inuit elder from Canada pointed out, this does not mean “that Inuit never planned for the future. [...] We are here today because our ancestors made sure that we could survive. They did not live one day at a time. They made us into human beings right from birth.” [39].

In recent anthropological studies in Greenland, the desire to plan ahead, to control or predict outcomes was described by women and elders as being a very Danish trait [40, 41]. Educational sessions that inform and prepare for parenthood could be perceived as a more Western approach. Nevertheless, having access to public services is important to participants and the possibility to attend MANU is appreciated. But talking to family members and friends with experience was most prominently described to be a common way to prepare for birth and parenthood. This approach is similar to statements in a qualitative study on child-rearing in Greenland from 1989 by L Zøllner [42], in which parents described not looking up information but rather following their instincts and traditions.

### Family involvement in parenthood and child-rearing

Ataatsimoorneq (community) as a value and providing toqqissisimaneq (security and care) are important aspects in child-rearing for the participants. Ataatsimoorneq and toqqissisimaneq are addressed in MANU within informational text or conversational exercises.

Toqqissisimaneq was defined as providing a secure home that is free of alcohol, hashish and violence and a home that is calm, stable, worry-free and has the child’s needs as a priority. Ensuring a secure and caring childhood is not an unusual desire in child-rearing. However, considering Greenland’s social challenges related to high misuse of alcohol and hashish resulting in adverse childhood experiences [43], it is striking that many participants name toqqissisimaneq and define it in this way. Some participants describe it in contrast to their own childhood experiences, while it is also important to know that these social challenges are a persistent focus within and about Greenland.

Cherishing ataatsimoorneq by dining together weekly with extended family members and that grandparents receive a significant role in child-rearing are Greenlandic traits also found in previous studies. Having a tight-knit network is common in Greenland. G Tróndheim [44] studied Greenlandic kinship and found that modern Greenlandic families still value and nurture familial relations and community, despite adopting more Western ways of individuality. Furthermore, a recent study in a smaller Greenlandic community found that familial and community connections play an important role in pregnancy decision making [15].

Ensuring a secure and caring environment while also cherishing community and family involvement can be challenging for new parents. In the transition to parenthood, parents re-identify themselves and find themselves in an ambivalence of how to detach themselves from their family, especially their own parents, while also seeking their support. Additionally, breaking free from one’s own adverse childhood experiences is challenging [45]. This can, for example, relate to participants weighing their dissatisfaction with the environment they were raised in while recognising how valuable the child-grandparent relation is to both the child and themselves. Finding one’s way as a parent, reflecting on one’s own childhood, identifying the valuable relations in one’s network, and experiencing ambivalence in one’s relations to one’s own parents are topics addressed in the MANU book. They are addressed as an overall topic within an informational text and a conversational exercise, but how much these topics are discussed in detail depends on the facilitator of the session and the individual parent.

Other values in child-rearing which participants mention are caring, respecting their child and setting boundaries to raise independent and confident children with respect and love for their fellow human beings. In Sami culture, for example, raising an independent human being who contributes to the community is also a core value in child-rearing [46]. In Arctic regions, like Sápmi (Norway) and in Nunavut (Canada), the raising of a capable human being is deeply connected to their ways of knowing and the involvement of elders (grandparents). Therefore elders, language and culture play a central role in the Indigenous-led parenting programmes in Sápmi (Norway) and Nunavut (Canada) [17, 19, 21, 47, 48]. The similarities of cultural values among Indigenous populations in the circumpolar region [15] suggest opportunities for learning from each other’s parenting programmes and point out the need for investigating family involvement in child-rearing in Greenland more closely.

### Opportunities for MANU

In the interviews, parents were able to provide suggestions and evaluate MANU’s material, delivery and accessibility. For a universal programme to be of value to parents, it should be accessible in a format and at a time that suits them [10].This study’s findings provide implications for: i) revising the MANU book’s format, ii) the opportunity to highlight how MANU creates a space for expectant and new parents to reflect, iii) reviewing how MANU sessions can be offered more conveniently to parents and iv) examining ways to engage and integrate men.

The MANU book was barely used outside the sessions even though participants found it to be a useful resource. In addition, many described using other informational resources such as smartphone applications, the internet and books on pregnancy, birth and parenthood. When considering revising the MANU book, it could be relevant to also draw inspiration from the Inunnguiniq parenting programme’s brochure [49] and the booklets on family connections, fatherhood, child-rearing and parenthood developed by the National Collaborating Centre for Indigenous Health and the Aqqiumavvik Society [48]. Additionally, briefer and more convenient access to preparational information could also be delivered through smartphone applications and phone messages, while taking into consideration that not everyone has a private (smart) phone, and internet access is limited in some regions in Greenland.

Listening to the thoughts and experiences of other participating parents in the MANU sessions was highly appreciated by interviewees. While the topics addressed were generally useful and relevant, participants suggested including more practical knowledge, for example, learning how to bathe or hold a newborn. Including such elements in the sessions would also address the participants’ feedback on the importance of experiencing an active session. Relevant components for this could be found in the Inunnguiniq parenting programme in Nunavut (Canada), where land components are included [21]. It would also be relevant to explore Indigenous methods for communicating information and facilitating discussions. This could be through storytelling and Sharing Circles.

In the interviews, parents discussed whether MANU sessions should be offered at a different time than during work hours in order to increase attendance of men in particular. Participants had no clear opinion or idea of when it would be better for them to attend, since those who already have children, would have to find childcare for when the sessions are taking place after work hours. In comparison, the Inunnguiniq parenting programme provides childcare as a part of the programme for parents to be able to attend the sessions [21]. However, this requires and depends on local resources and the organisation of MANU sessions.

Not being able to attend sessions due to work is not the only reason mentioned for men not participating. Participants described how men in general have more difficulties with expressing their thoughts and reflections, while this is a focal point of the MANU programme. Additionally, male participants expressed a need to talk to men in the same situation and found an organised fathers’ group to be potentially useful. MANU could consider providing fathers’ groups or divide parents into groups in sessions to meet this need. Integrating a land-based component could potentially motivate more men to participate and possibly stimulate them to converse.

Even though most participants had not attended all MANU sessions, the opportunity to do so was appreciated. Based on participants’ experiences, the sessions can be described as a space away from daily tasks – a space where expectant parents take the time as a couple to reflect and discuss. This is a valuable opportunity to reach out to parents and support them, making it even more important that local facilitators have the necessary resources to create such a space through MANU.

### Strengths and limitations

The broad number of participants from three different regions in Greenland and from both regional towns and smaller towns enabled a broad representation of parents, which supports internal generalisability [50]. Potential differences in participants’ perceptions based on location were not reported due to the small sample size and because adherence to the implementation of MANU varies between localities. Furthermore, participants’ reflections indicated that they had grown up with either different or similar resources. Finally, the authors have sought to present all perspectives equally to further strengthen internal generalisability of this paper [50]. Reaching such a high number of participants is, we suggest, due to collaborating with a Greenlandic interviewer (EJ), who engaged in recruitment, development of the interview guide and data collection. Collaborating with a Greenlandic interviewer also strengthened the validity of the study in two ways: First, the close collaboration and quality checks in translation and methods; secondly, by giving participants the opportunity to speak in their preferred language without interruptions, thereby ensuring more detailed responses.

The data collection was conducted during the first year of the COVID-19 pandemic. During that period, MANU sessions could not be offered as the programme intended, due to implemented restrictions to prevent infection. This has possibly influenced participants’ participation in and awareness of MANU. At the third study site, all participants had attended MANU, and more women than men had participated in this study. This could be a limitation regarding the degree of nuance in the presented findings.

## Conclusion

Based on the parents’ perspectives presented in this study, we conclude that parents’ notions and experiences of parenthood are generally addressed in the parenting programme MANU, but the experience and attendance of MANU depends on how it is organised and offered locally. The conversational exercises in MANU challenged parents’ notions of parenthood, but only in the sessions that parents attended, since barely anyone used the MANU book outside sessions. MANU has the possibility to create a space for parents to reflect and prepare. However, for MANU to be universal as intended and to reach both parents, the facilitation of sessions could be revisited, for example, with inspiration from other Arctic parenting programmes or making information accessible on multiple platforms to meet parents’ different ways of learning and accessing information. Next steps from this study are first to examine more deeply parents’ perspectives on the roles of their extended family in child-rearing and second identify the local opportunities and challenges of implementing MANU.

## Supplementary Information

**Additional file 1.**

**Additional file 2.**

## Data Availability

The datasets analysed during the current study are not publicly available due to the difficulty to de-identify qualitative data in a small population like Greenland, but data are available from the corresponding author on reasonable request.

## Abbreviations

CBPR: Community-based participatory research
MANU: Meeraq Angajoqqaat Nuannaarneq – the Greenlandic parenting programme
