# Supplementary material for: Parents’ perspectives on preparing for parenthood: a qualitative study on Greenland’s universal parenting programme MANU 0–1 year
Source: BMC Pregnancy Childbirth. 2022 Nov 20;22:859. doi: 10.1186/s12884-022-05170-4 (PMC9675961; doi:10.1186/s12884-022-05170-4)
Supplement: Supplementary file 1 — Additional file 1. [file 12884_2022_5170_MOESM1_ESM.pdf]

Christine Ingemann (chin@sdu.dk)

Parent interview guide

0. Introductory questions

0.1 How old are you?

0.2 Where were you born and raised?

0.3 Is this your first child? When was it born? How old are your other children?

0.4 Is it also your partner's first child?

0.5 On what grounds did you decide to have a child?

0.6 What in your life has changes since you have had a child? [OR] What changes in your life do you expect to happen when you have a child?

1. Family and parenting

1.1 How do you see your child grow up? Which values do you see important in child rearing?

1.2 Who in the family do you expect to play a role in your child's upbringing? Use the circle diagram to place from the middle and out the close to less close relations.

1.3 In your childhood - Did you feel attached to people from the older generation? E.g. grandparents or other Elders in your community who have or had a significant role for you?

1.4 What role did these people (e.g. grandparents) play in your upbringing?

1.5 How much do you want your parents or other family to be a part of your child's upbringing?

1.6 How many times do you meet in the family and what do you do together and are the children with?

1.7 Is parenting a thing between you, as parents, or a task for the whole family? Can you elaborate and give an example?

1.8 How will you use your own experiences from your upbringing in the upbringing of your child? What traditions from your family do you want to pass on to your own child?

1.9 What do you want to do differently compared to your own childhood and why?

1.10 When you have questions about becoming or being a parent - who do you turn to? Is it different in terms of subject? Give examples.

X. Rounding questions

X.1 What information, considerations or conversations do you see as important in order to best prepare yourself for parenthood? What would you like to have been better prepared for?

X.2 Do you have any last comment to this part?

2. MANU

2.1 Parent who did not participate in MANU

2.1.1 Have you been offered to attend MANU sessions? If yes, how, by whom and when? If no, can you describe what you have generally heard MANU is about?

2.1.2 Why did you choose not to participate in MANU?

Christine Ingemann (chin@sdu.dk)

2.1.3 Were you given the MANU book? Have you used and what do you think about it? Are there topics or exercises you find helpful?

2.1.4 During pregnancy, did you and your partner discuss things to prepare for when the baby arrives? E.g. upbringing or your relationship.

2.1.5 What preparations during pregnancy have been important to you? E.g. midwife consultations or information from family/friends.

2.1.6 Have you lacked any preparation, and if so, what?

2.2 Parents who have participated in MANU

2.2.1 When and how were you offered to attend MANU sessions?

2.2.2 Did your partner also participate in MANU? Why not/how was his/her experience?

2.2.3 What were your expectations to MANU?

2.2.4 How was it to attend MANU sessions?

2.2.5 How many times did you participate? (Why not all of them?)

2.2.6 How did you experience the MANU sessions? How is the session set-up and do you have suggestions for improvement?

2.2.7 Were you given the MANU book? Have you used and what do you think about it? Are there topics or exercises you find helpful?

2.2.8 Were there any topics that you thought about afterwards or that meant something to you?

2.2.9 During pregnancy, did you and your partner discuss things to prepare for when the baby arrives? E.g. upbringing or your relationship.

2.2.10 How did MANU help with possible considerations and reflections regarding pregnancy, birth and upbringing, which you yourself had thought of before or maybe not thought about?

2.2.11 Did MANU fit your expectations of a preparational program and views on parenthood and child rearing?

2.2.12 What preparations during pregnancy have been important to you? E.g. midwife consultations or information from family/friends.

2.2.13 Have you lacked any preparation, and if so, what?

X. Rounding questions

X.1 Do you have any last comments?

X.2 In case we should have any follow up questions, may we contact you again?
