# Supplementary figures and images for: Parents’ perspectives on preparing for parenthood: a qualitative study on Greenland’s universal parenting programme MANU 0–1 year
Source: BMC Pregnancy Childbirth. 2022 Nov 20;22:859. doi: 10.1186/s12884-022-05170-4 (PMC9675961; doi:10.1186/s12884-022-05170-4)

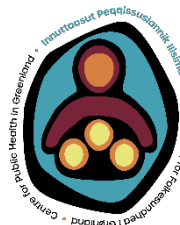

## Appendix 2. Circle diagram for interview guide.

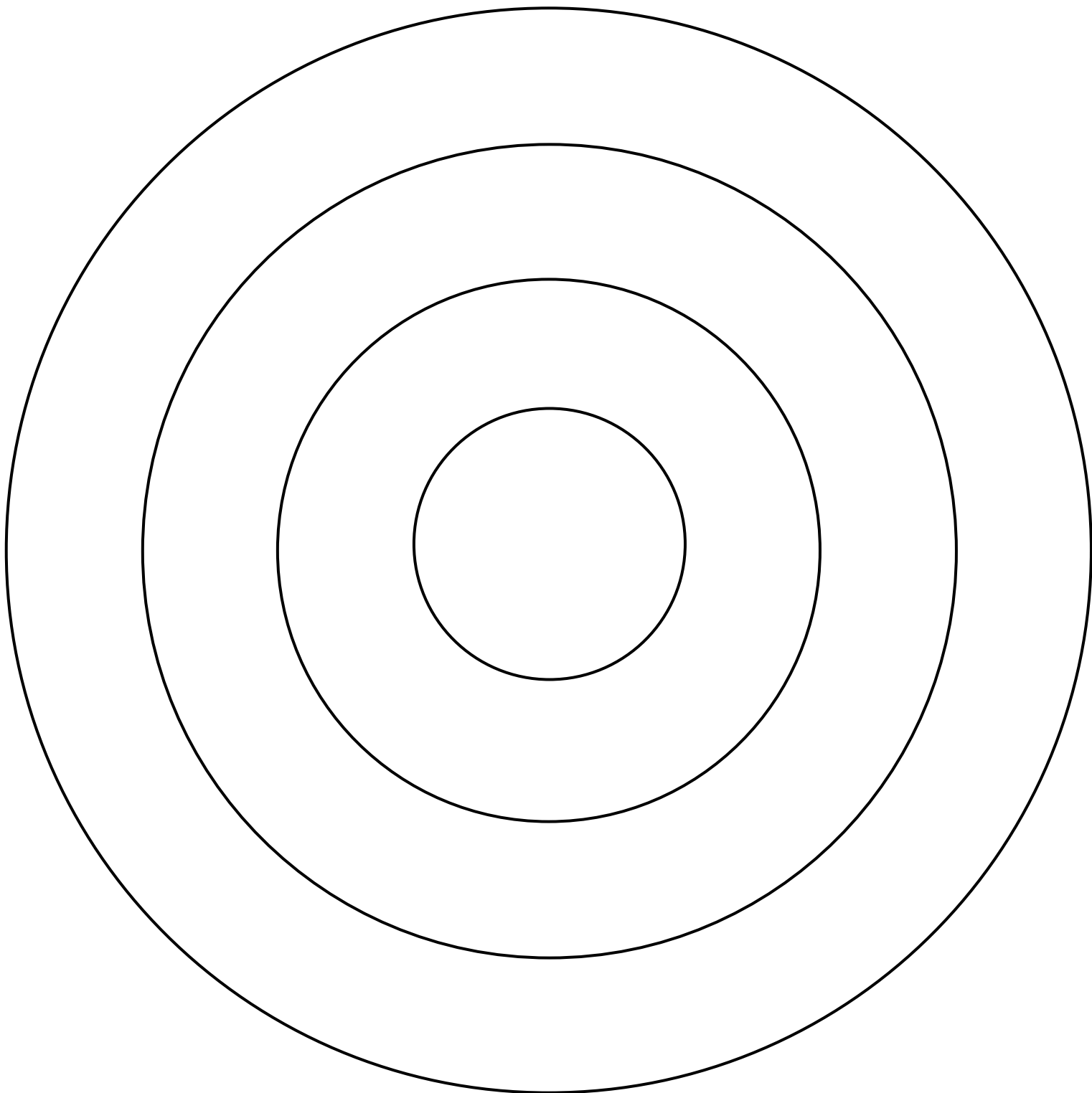

Supplement: Supplementary file 2 — Additional file 2. [file 12884_2022_5170_MOESM2_ESM.pdf]
